# Evaluation of some lignocellulosic byproducts of food industry for microbial xylitol production by *Candida tropicalis*

**DOI:** 10.1007/s13205-016-0521-8

**Published:** 2016-09-22

**Authors:** Kubra Eryasar, Seda Karasu-Yalcin

**Affiliations:** Food Engineering Department, Faculty of Engineering and Architecture, Abant Izzet Baysal University, Golkoy, 14280 Bolu Turkey

**Keywords:** *Candida tropicalis*, Xylitol, Chestnut shell, Hemicellulosic hydrolysate, Lignocellulosic wastes

## Abstract

Some lignocellulosic food byproducts such as potato peels, wheat bran, barley bran and chestnut shells were evaluated as potential sources of xylose for microbial xylitol production by yeasts. Potential yeast strains were selected after screening xylitol production of some indigenous yeasts in a defined fermentation medium. *Candida tropicalis* strains gave the highest results with 83.28 and 54.07 g/L xylitol production from 100 g/L xylose. Lignocellulosic materials were exposed to acid hydrolysis at different conditions. Chestnut shells gave the highest xylose yield and the hydrolysate of chestnut shells was used in further experiments in which xylitol productions of two potential *C. tropicalis* strains were investigated. Combined detoxification method including evaporation, overliming and activated charcoal with the use of threefold concentration and also yeast extract supplementation suggested to be efficient for both growth and product formation in chestnut shell hydrolysate in which 40 % xylitol yield was obtained. It was concluded that detoxified and fortified chestnut shell hydrolysate could be a potential medium for xylitol production.

## Introduction

Xylitol is a naturally occurring five-carbon sugar alcohol that is used commercially as a natural sweetener in various food products. It is principally used in certain sweetened products such as confectionery, in personal health products such as mouthwash and toothpaste, and in the pharmaceutical industry such as a sweetener or coating agent for pharmaceutical products (Rafiqul and Mimi Sakinah 2013). Commercial chemical production of xylitol is based on hydrogenation of xylose in a nickel-catalysed process which is an energy and cost-demanding. Therefore, it is known that some alternative biotechnological processes have been studied, especially those involving yeasts from *Candida* genus (Ur-Rehman et al. 2015). Xylitol is an intermediate metabolite of xylose utilization by microbial strains. Numerous microbial species have a metabolic system with NADPH-dependent xylose reductase and NAD^+^-dependent xylitol dehydrogenase enzymes which are induced by xylose. *Candida tropicalis*, *Candida guilliermondii*, *Candida athensensis*, *Candida parapsilosis* and *Debaryomyces hansenii* are among the yeast species reported to produce high yields of xylitol (Albuquerque et al. 2015b; Mohamad et al. 2015).

Lignocellulosic materials are widespread, abundant, renewable, cost effective and economical sources of polysaccharides which can be used for xylitol production. Residues of some agricultural and food industries contain lignocellulose as organic matter which is mainly composed of cellulose, hemicellulose, lignin and smaller amounts of pectin, protein and ashes. Hemicellulose, which is not chemically homogenous, comprises of pentoses (xylose, arabinose), hexoses (mannose, glucose, galactose), and sugar acids (Ur-Rehman et al. 2015). After pretreatment with acid, alkali and/or enzymes, the carbohydrate fraction of the plant cell wall can be converted into monomeric fermentable sugars (Chandel et al. 2013). Xylose is a major sugar derived from the hydrolysis of lignocellulosic biomass, among the other sugars, such as mannose, galactose, arabinose, and rhamnose. Acid hydrolysis using diluted concentrations of acids is a very widespread treatment due to its efficiency in the process of obtaining sugars (Albuquerque et al. 2015b). However, products that are considered toxic for microbial growth, such as phenolic compounds, furfural, 5-hydroxymethylfurfural, acetic acid, and formic acid are formed (Albuquerque et al. 2015b; Mustapa Kamal et al. 2011; Mateo et al. 2014). Several detoxification methods have been reported to be effective in reducing the inhibitors. The most common methods employed are; neutralization and overliming, biological adaptation, extraction with organic solvent, adsorption with activated charcoal and ion-exchange resins. The effectiveness of the methods depends on the types of hemicellulosic hydrolysate and the species of microorganisms employed because different types of hydrolysate have different degrees of toxicity and each microorganism with different degrees of tolerance to inhibitors (Mustapa Kamal et al. 2011).

The aim of this study was to investigate potential use of some hemicellulosic hydrolysates prepared from lignocellulosic byproducts as sources of xylose for xylitol production by indigenous yeast strains.

## Materials and methods

### Yeast strains

Sixteen foodborne yeast strains were obtained from Department of Food Engineering, Hacettepe University (Ankara, Turkey) with kind supports of Professor Dr. Z. Yesim Ozbas. Most of these indigenous yeasts were cheese-originated as shown in Table 1 and had been isolated and identified as described by Senses-Ergul and Ozbas (2006) and Karasu-Yalcin et al. (2012). The yeast strains were activated in yeast extract malt extract (YM) agar and kept at +4 °C as stock cultures.

**Table 1:** Isolation sources of the indigenous yeast strains and obtained maximum xylitol concentrations in defined fermentation medium containing 100 g/L xylose

Yeast strain	Isolation source	Maximum xylitol concentration (g/L)
*Candida tropicalis* M2	Mihalic cheese	83.28
*Candida tropicalis* M43	Mihalic cheese	54.07
*Candida tropicalis* M55	Mihalic cheese	20.72
*Candida famata* M4	Mihalic cheese	7.83
*Candida famata* M58	Mihalic cheese	2.04
*Candida famata* T52	Erzincan tulum cheese	4.32
*Candida famata* T7	Erzincan tulum cheese	7.77
*Candida famata* M41	Mihalic cheese	9.28
*Candida famata* T98	Erzincan tulum cheese	9.66
*Candida famata* T169	Erzincan tulum cheese	15.93
*Candida famata* M51	Mihalic cheese	1.65
*Candida famata* M5	Mihalic cheese	2.80
*Candida famata* M92	Mihalic cheese	18.37
*Candida famata* M89	Mihalic cheese	8.40
*Debaryomyces hansenii* 31/2	Honey	8.65
*Candida guilliermondii* M54	Mihalic cheese	0.19

### Growth and fermentation media

The inocula used in the experiments were prepared by incubation of the cultures in water bath shaker at 30 °C and 90 strokes/min for 24 h in a growth medium containing xylose, 50; yeast extract, 3; pepton, 2; (NH_4_)_2_SO_4_, 0.5; KH_2_PO_4_, 2; MgSO_4_·7H_2_O, 1 (Azuma et al. 2000).

For screening xylitol production of the yeast strains, a defined fermentation medium including (g/L): xylose, 100; yeast extract, 3; pepton, 2; (NH_4_)_2_SO_4_, 0.5; KH_2_PO_4_, 2; MgSO_4_·7H_2_O, 1 was used (Azuma et al. 2000).

### Lignocellulosic wastes and byproducts

Chestnut shells were obtained from Kafkas Company (Bursa, Turkey) as a waste of candied chestnut production. Potato peels were obtained from Bolpat Company (Bolu, Turkey) as a waste of potato wedges. Barley bran was provided from Field Crops Research Institute (Ankara, Turkey) as a byproduct of hull-less barley milling. As another byproduct, wheat bran was obtained from the millers of Gulen Flour Company (Bolu, Turkey).

### Equipment and fermentation conditions for xylitol production

Xylitol production experiments were carried out in water bath shakers at 30 °C and 100 strokes/min shaking rate. Fermentation media were inoculated at a ratio of 5 % (v/v). Initial pH of the media was adjusted to 6.25.

### Preparation of hydrolysates

Potato peels were dried in an incubator at 60 °C for 12–15 h and then cut by a blender. Chestnut shells were grinded in a laboratory miller (Retsch ZM 200) after drying at the same conditions. Wheat and barley bran were used directly in hydrolysis. The pretreated byproducts were separately exposed to sulfuric acid (1.25, 2.5, 5 and 10 %) using three different solid/liquid ratios (5, 8, and 10 g/100 mL). The mixtures were kept in autoclave at 121 °C for 60 min, and then centrifuged at 7700×*g* for 15 min. The supernatant was used as the raw hydrolysate.

### Pretreatment experiments on chestnut shell hydrolysate for achieving xylitol production

According to the xylose concentrations of the raw hydrolysates, chestnut shells were chosen for further experiments. Some detoxification steps were employed on chestnut shell hydrolysate, followed by concentration and also nutrient supplementation.

First, xylitol productions of *C. tropicalis* M2 and *C. tropicalis* M43 were examined in the raw chestnut shell hydrolysate (H) without any detoxification. The hydrolysate was then treated with activated charcoal (powder form) at a ratio of 5 g/100 mL in a water bath shaker at 30 °C and 200 strokes/min for 1 h. After coarse filtration, the detoxified clear hydrolysate (H1) was obtained. The third medium (H2) was prepared by the addition of glucose (8 %) to detoxified hydrolysate. This medium was also supplemented with yeast extract (1.5 g/L) in addition to glucose for preparation of medium coded H3.

In the second stage of the experiments, the hydrolysate was concentrated under vacuum evaporator at 75 °C after activated charcoal treatment and used without nutrient supplementation. Concentration was employed leading to threefold (H4), fourfold (H5), and sevenfold (H6) decreases in volume.

Medium H7 was prepared using a modified method including different detoxification steps as well as concentration (Canilha et al. 2005; Baek and Kwon 2007; Ramesh et al. 2013). The raw hydrolysate was kept at 100 °C for 15 min to remove volatile toxic compounds. After that pH of the hydrolysate was increased to 10.0 (overliming) by calcium hydroxide and the pellet formed was removed by coarse filtration. The pH then decreased to 7.0 using 13 M sulfuric acid and centrifuged at 7700×*g* for 15 min. The supernatant was used in activated charcoal treatments. The hydrolysate was exposed to activated charcoal at a ratio of 5 g/100 mL in a water bath shaker at 30 °C and 200 strokes/min for 1 h. After coarse filtration, the detoxified clear hydrolysate was obtained. The hydrolysate was then concentrated under vacuum evaporator (until threefold decrease in volume) at 75 °C.

The medium H7 was also supplemented with xylose and 1.5 g/L yeast extract. Initial xylose concentrations were adjusted 49 g/L (H8) and 94 g/L (H9) in the media by evaporation and also xylose addition when needed.

Sterilizations of all of the prepared media were done by filtration after their pH was adjusted to 6.25.

### Measurement of yeast growth

Microbial growth was measured by determining yeast cell number. During fermentation in chestnut shell hydrolysate, samples were taken from the media at specific time intervals. Cultures were spread on YM agar after preparing serial dilutions of the culture, and incubated at 28 °C for 48 h. Yeast count was determined in terms of cfu/mL.

### Analytical methods

Xylose analysis was performed using d-xylose enzymatic test kits (Megazyme Assay Kits, Megazyme International Ireland Limited, Wicklow, Ireland). Xylitol analysis was performed using d-sorbitol/xylitol enzymatic test kits (Megazyme Assay Kits, Megazyme International Ireland Limited, Wicklow, Ireland). Amount of total phenolic compounds was measured using Folin-Ciocalteu method (Singleton and Rossi 1965). Total nitrogen content of chestnut shell hydrolysate was measured by Kjeldahl method.

## Results and discussion

### Selection of yeast strain

Maximum xylitol concentrations obtained for different indigenous yeast strains in the defined fermentation medium were given in Table 1. The highest xylitol concentration (83.28 g/L) was obtained for *C. tropicalis* M2, followed by *C. tropicalis* M43 (54.07 g/L). It was found that *C. tropicalis* M55, *C. famata* M92, and *C. famata* T169 also have potential for xylitol production. *C. tropicalis* was also the subject of some other studies suggested as the best xylitol-producing species (West 2009; Mello Lourenco et al. 2014). Xylitol yield (83 %) of cheese-originated M2 strain was promising when compared with the results obtained by the other yeast strains in the reported studies. Mello Lourenco et al. (2014) investigated the potential of 28 yeast isolates originating from sugarcane filter cake for bioconversion of d-xylose to xylitol. It was reported that xylitol yields ranged from 6 to 61 % and the highest was presented by a *C. tropicalis* strain which produced 32.97 g/L xylitol from 50 g/L xylose. West (2009) screened xylitol production capabilities of five known xylitol-producing strains in a grass hydrolysate and reported that the highest xylitol level (approximately 17 g/kg) was obtained by a *C. tropicalis* strain.

In strain-screening studies, usually strains originated from lignocellulosic sources, i.e. forestry residues, are used (Mello Lourenco et al. 2014; Kamat et al. 2013; Guamán-Burneo et al. 2015). It was reported that the yeasts adapted to these environments were expected to have high ability to convert xylose to xylitol. Apart from that our study revealed that yeasts coming from food sources other than lignocellulosic ones could be efficient for this purpose. Kamat et al. (2013), who studied with isolates from mangrove forests, reported that the high salt concentration and low water potential of such ecosystem favour the growth of microbes that can maintain a lower water potential than the surrounding saline waters. The related fungi maintain this gradient by intercellular accumulation of polyols, such as glycerol, mannitol, sorbitol, and xylitol. This can be correlated to high xylitol production of *C. tropicalis* M2 as well as *C. tropicalis* M43 in this study as a result of adaptation to high salt concentration, since their origin Mihalic cheese is one of the most salty cheeses of Turkey.

### Use of different lignocellulosic byproducts

Xylose concentrations obtained in different raw hydrolysates (without detoxification and concentration) prepared using various sulfuric acid concentrations and solid/liquid ratios were represented in Table 2. It was found that the highest xylose concentrations were obtained in chestnut shell hydrolysate followed by potato peel hydrolysate. Xylose concentrations increased with the increase in solid/liquid ratio especially in chestnut shell hydrolysate. The solid/liquid ratios higher than 10 g/100 mL were also examined but could not be used because of increased water-binding activity of the chestnut shells. The highest xylose concentration in raw chestnut shell hydrolysate was 8.33 g/L for 10 g/100 mL solid/liquid ratio and 10 % sulfuric acid. However, similar results were obtained when different sulfuric acid concentrations were used for chestnut shell hydrolysate as well as for the others.

**Table 2:** Xylose concentrations (g/L) obtained after acid hydrolysis for different hemicellulosic byproducts

Solid/liquid ratio (g/100 mL)	Sulfuric acid concentration (%)			
	1.25	2.5	5	10
Chestnut shells				
5	4.22	2.61	3.90	2.33
8	5.13	4.38	3.11	4.81
10	8.23	7.89	8.01	8.33
Potato peels				
5	2.35	3.68	1.78	2.87
8	2.67	4.41	3.19	2.48
10	4.67	5.41	3.19	3.48
Wheat bran				
5	0.15	0.26	0.21	0.17
8	0.26	0.52	0.33	0.45
10	0.89	1.67	0.88	0.91
Barley bran				
5	1.67	1.56	1.02	2.06
8	2.45	1.93	2.30	2.18
10	2.34	2.05	3.65	2.87

According to the obtained xylose concentrations, chestnut shell was chosen as a potential hemicellulosic material for xylitol production. It was demonstrated that 10 g/100 mL solid/liquid ratio and 1.25 % sulfuric acid would be appropriate for initial preparation of the hydrolysate before detoxification and concentration. Various lignocellulosic residues have been reported to be used for xylitol production, some of which were corncob (Cheng et al. 2009; Kamat et al. 2013; Ramesh et al. 2013) sugarcane straw (Hernández-Pérez et al. 2016), cotton stalks (Akpinar et al. 2011), grape marc (Salgado et al. 2012), cashew apple bagasse (Albuquerque et al. 2015a), wheat straw (Canilha et al. 2005), wood sawdust (Rafiqul and Mimi Sakinah 2012), vine trimming wastes (Rivas et al. 2007), and rice husks (Rambo et al. 2013). Chestnut shells have been used in some studies for different purposes (Aires et al. 2016). Total amount of cellulose and hemicellulose in chestnut shell was reported to be 48.5 % by Gómez et al. (2005). It was used for the first time for xylitol production in this study. Acid hydrolysis conditions recommended were similar to those suggested by Baek and Kwon (2007) which were 1.5 % sulfuric acid and 1:10 solid to liquid ratio. Miura et al. (2011) also studied different hydrolyzation conditions for hydrolysis of *Sasa senanensis* culm and suggested the use of 2 % sulfuric acid and 5 g/g liquid to solid ratio. Biotechnological evaluation of chestnut shells would be valuable because yet there is not a useful employment of this material in industry. Turkey is one of the biggest chestnut producers in the world, leading to its various industrial applications and also high amounts of shells (Karadeniz 2013).

### Xylitol production in chestnut shell hydrolysate after various pretreatments

It is known that xylitol production is affected by the remaining inhibitors in the hemicellulosic hydrolysates and sensitivity to these inhibitors could be strain specific. For this reason, xylitol production in the prepared chestnut shell hydrolysate was investigated for the two promising strains, *C. tropicalis* M2 and *C. tropicalis* M43. Growth and xylitol production capabilities of the two strains in ten different prepared media were represented in Table 3. Although xylitol yield obtained with *C. tropicalis* M2 was considerably higher than that of *C. tropicalis* M43 in the defined medium, similar results were obtained in chestnut shell hydrolysate for the two strains, leading to different sensitivity of the strains to the inhibitory substances in the hydrolysate medium (Mustapa Kamal et al. 2011).

**Table 3:** Growth and xylitol production capabilities of *C. tropicalis* M2 and *C. tropicalis* M43 in the media prepared from chestnut shell hydrolysate exposed to different treatments

Media	*C. tropicalis* M2		*C. tropicalis* M43	
	Cell count increase [log (cfu/mL)]	Xylitol concentration (g/L)	Cell count increase [log (cfu/mL)]	Xylitol concentration (g/L)
H	0.9	–	1.1	–
H1	1.1	0.8	1.7	0.7
H2	2.1	1.3	2.2	1.5
H3	2.8	1.5	2.9	1.8
H4	2.2	4.4	2.7	4.9
H5	2.9	2.1	2.5	3.2
H6	–	–	–	–
H7	3.1	6.1	2.9	5.8
H8	2.1	19.6	2.0	19.9
H9	3.0	27.7	2.6	25.3

It was found that yeast growth was at low levels and xylitol production did not occur in the raw hydrolysate without any detoxification or nutrient supplementation. Activated charcoal treatment caused a little increase in xylitol production and yeast growth for both of the strains in medium H1. Addition of glucose to medium H1 enhanced cell growth but obtained xylitol concentrations were again very low. There have been contradictory reports existed for the fermentation of xylose to xylitol by yeasts when glucose was added as a cosubstrate. It was reported that although certain amounts of glucose supplementation improved overall process, higher amounts reduced xylose consumption rate and xylitol productivity. In addition, glucose is known to be utilized for cell growth faster than xylose was used, allowing NADPH regeneration by metabolism through the pentose phosphate pathway (Parajó et al. 1998). Tamburini et al. (2010) reported that the addition of glucose as cosubstrate to xylose-containing medium caused an increase in biomass yield, but a delay in xylitol production; because xylose utilization took place only when the glucose had been completely metabolized.

Total nitrogen content of chestnut shell hydrolysate was very low (0.025 %) and fortification of the hydrolysate with a nitrogen source would be appropriate for enhancing growth and product yield. Positive effect of glucose and yeast extract addition especially on yeast growth can be seen from the results of medium H3, with a little increase in xylitol production. There are some reported studies suggesting nutrient supplementation to hemicellulosic hydrolysates, i.e. yeast extract in hardwood waste hydrolysate (Ko et al. 2008), rice bran extract in wheat straw hydrolysate (Canilha et al. 2005), urea in cashew apple bagasse hydrolysate (Albuquerque et al. 2015a), yeast extract in corncob hydrolysate (Ramesh et al. 2013), and yeast extract and ammonium sulphate in grass hydrolysate (West 2009).

Preparation of the media H4, H5, and H6 included concentration instead of nutrient supplementation. Concentration level was one of the most important parameters in the hydrolysate treatment. It can be observed that threefold concentration for preparation of medium H4 made better effects on xylitol production other than glucose addition. Medium H4 contained 15 g/L xylose and gave 32.6 % xylitol yield. Xylitol yield decreased with increasing concentration degree in Medium H5 containing 27.5 g/L xylose although cell growth was better especially for the strain M2. Xylose concentration of medium H6 reached to 66.7 g/L, but results obtained for this medium demonstrated adverse effect of high concentration degrees, in which no growth and product formation occured. It was estimated that this was because of increased level of toxic compounds after sevenfold concentration. In addition to known inhibitory effects, Rafiqul et al. (2015) reported that the enzyme xylose reductase was inhibited by the toxic compounds in the hemicellulosic hydrolysates. Canilha et al. (2005) also demonstrated the negative effects of high concentration levels for wheat straw hemicellulosic hydrolysate. It was reported that three-, four- and fivefold concentrations were used and the best conditions to perform the bioconversion consisted in using a threefold concentrated hydrolysate supplemented with ammonium sulphate and rice bran extract. However, higher concentration levels could be used in different hemicellulosic hydrolysates as reported in some studies (Salgado et al. 2012; Carvalho et al. 2006; Mateo et al. 2013). In this study, threefold concentration was recommended and used in the experiments including combined detoxification methods such as evaporation, overliming and activated charcoal.

Combining various detoxification methods such as evaporation, overliming, activated charcoal treatment with appropriate concentration was suggested to be the best way for xylitol production from chestnut shell hydrolysate. These methods were used in the preparation of H7, H8, and H9 which gave the highest results for xylitol production. By detoxification process used in the preparation of H7, H8, and H9, amount of total phenolic compound in the hydrolysate was considerably reduced. Total phenolic compound content decreased to 286.2 mg/L after overliming and pH adjustment, while it was 2150.3 mg/L in raw hydrolysate. It was found as 18.9 mg/L after activated charcoal treatment, but increased to 39.2 mg/L after concentration and resulted in 98 % reduction in total phenolics. Maximum xylitol concentrations of 6.1 and 5.8 g/L were obtained leading to 41 and 39 % product yield in the medium H7, for the strains M2 and M43, respectively. A considerable increase in xylitol production occured by the fortification of detoxified and concentrated hydrolysate with xylose and yeast extract. In the hydrolysate containing 94 g/L xylose (medium H9), maximum xylitol concentration was obtained as 27.7 g/L for *C. tropicalis* M2, while it was 25.3 g/L for *C. tropicalis* M43. Although obtained xylitol concentrations were low because of low xylose concentration (15 g/L), approximately 40 % xylitol yield was promising for both of the strains in the medium H7. Media H8 and H9 were used just for testing the effects of high xylose concentrations in the hydrolysate. It was found that 49 g/L initial xylose concentration would be more advantageous for xylitol yield, which could also be reached by high concentration levels. It is thought that xylose concentration of detoxified chestnut shell hydrolysate could be increased by increasing the concentration level more than threefold if only initial preparation (acid hydrolysis) and detoxification (activated charcoal and overliming) steps are optimized.

In conclusion, this study revealed that fortified and detoxified chestnut shell hemicellulosic hydrolysate could be evaluated as a novel fermentation medium for xylitol production by *C. tropicalis* M2 as well as by *C. tropicalis* M43. Optimization of preparation and detoxification steps was suggested to improve xylitol yield.
